# Fibroepithelial Polyp in a Duplicated Ureter

**DOI:** 10.5334/jbsr.2232

**Published:** 2020-09-22

**Authors:** Jae Young Lee, Seung Soo Kim, Chan Ho Park

**Affiliations:** 1 Soonchunhyang University College of Medicine, Cheonan Hospital, KR

**Keywords:** ureter, fibroepithelial polyp, computed tomography, urography

## Abstract

**Teaching point:** A fibroepithelial polyp is an intraluminal, long, slender neoplasm that frequently occurs in the ureteropelvic junction and upper ureter.

## Case History

**Figure 1:**
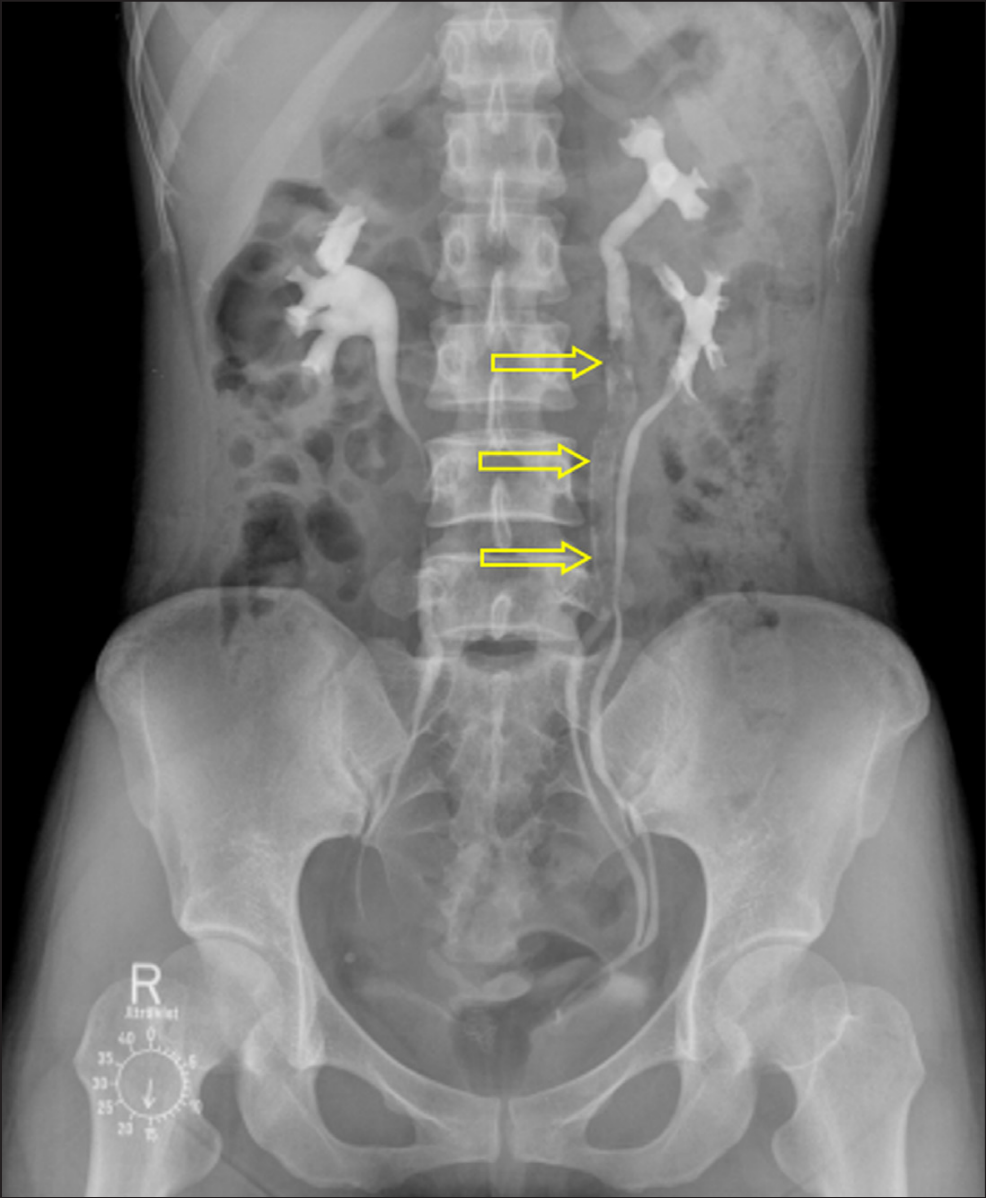


**Figure 2:**
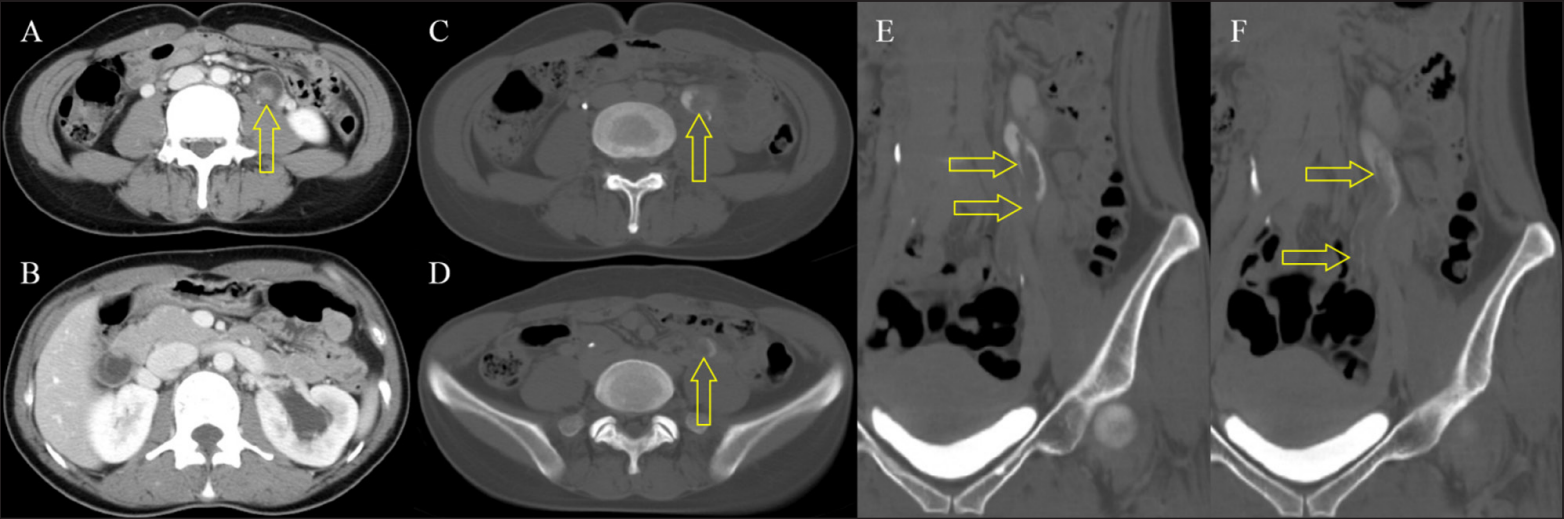


**Figure 3:**
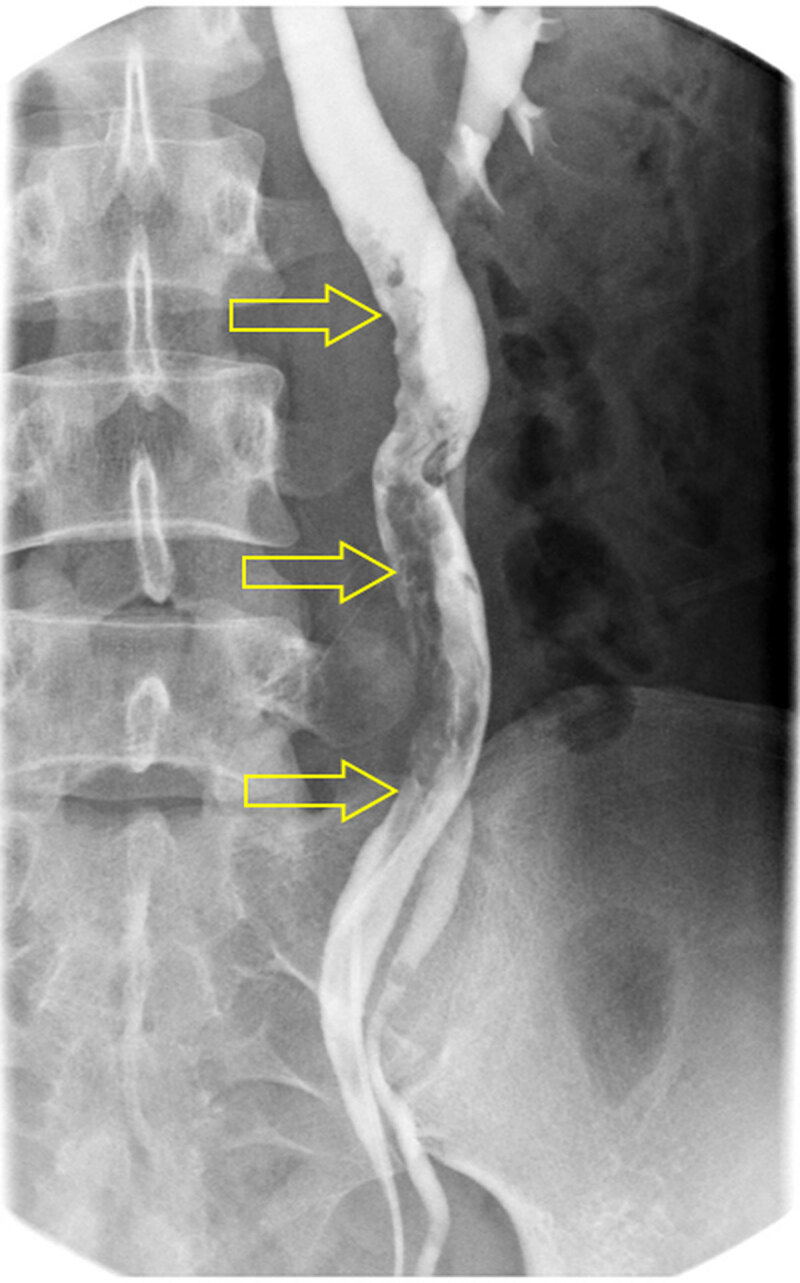


A 36-year-old female was admitted to our hospital with hematuria and left flank pain. Her past medical history was unremarkable. Urine analysis showed >100 leukocytes and 5–9 erythrocytes per high-power field. Intravenous urography (Figure 1) revealed incomplete duplication of the left ureter with a 9-cm-long filling defect (open arrows) in the upper moiety. Contrast-enhanced computed tomography (CT) was performed, and axial nephrographic phase CT images (Figure 2A and 2B) showed an enhancing mass (open arrow) in the left upper ureter, with ipsilateral mild hydronephrosis. Axial and coronal reformatted excretory phase CT images (Figure 2C–F) demonstrated an intraluminal mass (open arrow) filling the left upper ureter. Contrast material flowed around the mass (open arrow). Retrograde pyelography (Figure 3) confirmed along and irregular filling defect (open arrows) in the left ureter. The patient underwent left ureterostomy and excision of the mass, and was diagnosed with fibroepithelial polyp (FEP).

## Comment

Ureteral FEP is a rare benign tumor that consists of a fibrovascular stromal core covered by transitional epithelium. It is the most common benign, non-epithelial, ureteral neoplasm and predominantly presents in the third to the fifth decades of life. Although the exact etiology of FEP is unclear, congenital factors and chronic inflammation have been proposed. FEPs can develop in any part of the urinary tract, but most occur in the ureter, particularly in the ureteropelvic junction or upper ureter. The majority of patients with FEP demonstrate hematuria and/or flank pain. FEP is usually smaller than 5 cm, but a 14 cm polyp has been reported [1]. Most FEPs are solitary, but multiple or bilateral occurrences have been reported [1].

On imaging, FEP is frequently mistaken for urothelial carcinoma, which is the most common neoplasm in the urinary tract. Although the imaging features of FEP are variable, it usually manifests as a long, slender, and smooth ureteral filling defect on urography images. Compared to urothelial carcinomas, FEPs occur in younger patients. In addition, FEPs occasionally show mobility, unlike the fixed urothelial carcinomas. Nevertheless, it is difficult to pre-operatively differentiate FEP from urothelial carcinoma due to the rarity of FEP and the overlap in clinical presentations and radiological findings. The treatment of choice for FEP is complete excision [1].

## Author Information

On a title page separate from the manuscript.
